# Examples of Reliability Models of a Renewable Technical Object in Relation to Special Vehicles

**DOI:** 10.3390/ma18153552

**Published:** 2025-07-29

**Authors:** Michał Stawowiak, Aleksander Gwiazda, Santina Topolska, Małgorzata Olender-Skóra

**Affiliations:** 1 Department of Mining Mechanization and Robotization, Faculty of Mining, Safety Engineering and Industrial Automation, Silesian University of Technology, Akademicka 2, 44-100 Gliwice, Poland; 2 Department of Engineering Processes Automation and Integrated Manufacturing Systems, Faculty of Mechanical Engineering, Silesian University of Technology, Konarskiego 18A, 44-100 Gliwice, Poland; aleksander.gwiazda@polsl.pl; 3 Department of Welding Engineering, Faculty of Mechanical Engineering, Silesian University of Technology, Konarskiego 18A, 44-100 Gliwice, Poland; santina.topolska@polsl.pl

**Keywords:** model, repair, reliability, restoration, special vehicle

## Abstract

The article describes examples of reliability models of a renewable technical object. The proposed models are mathematical models that, according to the author, are best suited to presenting problems resulting from the operation of the analyzed technical objects. These objects are special vehicles, in this case garbage trucks with plate compaction and rear loading of waste containers. The author described two models: one where a model was analyzed and the replacement of a worn part with a brand new part was assumed, and a model where the worn element was repaired (renewed), so that after the repair, the element showed features as if it were a brand new element. Each of the examples was considered based on operational data from city cleaning companies. Data obtained from service books was used for calculations. The analyzed examples are concluded with short conclusions. In turn, the entire article ends with a summary in the form of conclusions resulting from the use of these specific models. The author draws attention to the reasonableness of their use in the scope analyzed by him, and the benefits that result from the use of these models.

## 1. Introduction

Due to the complexity of their construction, special vehicles are fascinating technical objects in terms of the process of wear of their components. To properly present and match the appropriate model to a given process of wear of a specific component or subassembly, which is part of a special vehicle, it is necessary to consider the mechanism of wear of this component. This mechanism very often results from the nature of the work of such components and the environment in which they work. The elements and subassemblies in question are divided into the following:

(a) elements working until first failure,
(b) repairable elements that regain their properties and nominal technical parameters after repair.

It is worth noting here that the vast majority of special vehicles are technical objects that are renewable objects. The renewal of a technical object, such as a special vehicle, can be of a different nature. The procedures that stand out are as follows:

(a) Post-damage restoration;
(b) Preventive (pre-emptive) renewal.

Renewal can have various effects, namely, the following:

(a) We replace the damaged element or the entire technical object with a new one;
(b) A damaged element or an entire technical object is repaired.

The result of the repair may be the following:

(a) Complete restoration of the element’s ability to perform its functions.
(b) Partial restoration of the ability of the element to perform its functions.

According to the author, the models presented above are best suited for the analysis of the elements under consideration. In turn, the operational data used to present the mathematical models described in the further part of the article come from operational practice—repair shops, which constitute the technical and repair facilities of city cleaning plants located in different regions of the country.

Referring to the above considerations regarding the repair or replacement of damaged components of special vehicles, the author refers to a whole group of components that influence the maintenance of the rated parameters of a technical vehicle so that it can perform its assigned tasks in municipal services. As an example, described later in the article, the issue of lifting containers with waste and then compacting it within the loading chute of a garbage truck can be discussed. This directly impacts the garbage truck’s efficiency, measured in tons of waste collected per hour. Weakening or complete failure of the above-mentioned components results in a drastic reduction in the amount of waste the garbage truck should transport out of the city within an hour or shift.

In this article, the author focused on post-damage renewal models, sometimes called emergency, although the concept of failure is not included in technical standards and is rather used in everyday language. The omission of the topic of preventive renewal results from the fact that it usually includes economic aspects, which are the basic criterion for choosing a specific strategy. In the scope of considerations of this article, economic issues are omitted. Certain issues of preventive renewal were presented in the work [1]; however, the basic work in this area can be considered [2].

The reliability of a technical object is its ability (feature, property) to meet the requirements placed on it.

Renewal theory is a branch of probability theory that generalizes Poisson processes to those in which the intervals between events (here called renewals) have an arbitrary distribution.

In the literature [3], the authors addressed the cost issues associated with the transportation and separation of municipal waste. To address this issue, this paper considers two models. The first model utilizes the vehicle routing problem (VRP) to direct fleets between waste generation and separation facilities. The second model aims to allocate resources from waste separation facilities to a set of recovery facilities or landfills. To the authors’ knowledge, most previous research on this topic has focused exclusively on deterministic implementations. Furthermore, recent studies typically focus on uncertain parameters in the area of waste generation. Furthermore, several related studies have developed an uncertain parameter model that focuses on facilitating separation. This study considers the uncertain parameters of separation facility performance, as well as the importance of recovering value from each container, with the goal of increasing operational efficiency.

In the literature [4], the authors addressed the specific problem of optimizing the routes of waste collection and transport vehicles, which arise during the collection and disposal of bulky recyclable waste. Containers of various types, used to store various waste materials, must be collected, emptied at appropriate disposal facilities, and replaced with empty containers when full. All orders must be processed, and the routes of these vehicles are subject to maximum duration constraints. The primary objective is to minimize the number of vehicles, while minimizing the total route duration is a secondary objective. The problem belongs to the class of roll-on-roll-off vehicle routing problems (RR-VRP), although certain features of the case study, such as the free circulation of containers and the limited availability of spare containers, allow us to use them in the approach to the proposed solution.

In [5], a real-world problem of waste collection vehicle routing with time windows (VRPTW) is considered, taking into account multiple trips and drivers’ lunch breaks. The well-known Solomon insertion algorithm extends to this problem. Although minimizing the number of vehicles and the total driving time is the main goal of vehicle routing problems in the literature, this paper also considers the routing and workload balance of the proposed solution, as these are very important aspects in practical applications. To improve the routing and workload balance, a VRPTW algorithm for waste collection based on capacitive clustering is developed. The proposed algorithms were successfully implemented and deployed to real-world waste collection problems at Waste Management, Inc. The paper also presents a set of benchmarks VRPTW problems for waste collection.

The article [6] examines waste management problems in urban areas and analyzes the systems used in this area. The first part of the article focuses on waste collection and transportation activities in urban systems, as well as on understanding the causes of inefficiency of traditional waste collection systems. A prototype of an intelligent waste collection container is presented, aimed at achieving more efficient waste collection, combined with the use of modern information and communication technologies in waste collection and transportation systems. The waste collection problem is defined as a dynamic CVRP. To solve this problem, a heuristic-based methodology is proposed that allows for the determination of optimal dynamic routes, minimizing total costs, i.e., transportation costs.

The aim of this article is to propose selected mathematical models that allow for estimating with high accuracy the period of time over which a selected element will fail or the number of elements that should be kept in the warehouse to ensure the continuous operation of a special vehicle. The authors believe that the content of this article ideally fills a research gap in this area. A thorough literature review reveals a lack of studies addressing the reliability of special-purpose vehicles, particularly those used for routine urban maintenance. A research problem stemming from the limited number of studies in this area is the inability to reference and correlate the obtained results with those from other studies addressing the reliability of special-purpose vehicles. However, the authors plan further research on components susceptible to failure in special-purpose vehicles.

## 2. Materials and Methods

The renewal model described by the author using the reliability theory is based on parameters generally available in the world literature relating to this type of model, based on actual numerical data, as presented in the examples later in the article.

The operational data used to write this article and present the examples presented here comes from industry, specifically from a company involved in urban sanitation. The authors were very keen to obtain real-world data to support the discussion described later in the article. The results obtained are consistent with operational practice and were consulted with maintenance services, service technicians, and municipal equipment manufacturers. The data used for the calculations was collected by the maintenance services at the selected urban sanitation company. This data enabled the construction of graphs illustrating the wear and tear of the components described in the apron garbage trucks. This data covers a period of more than ten years, allowing for a comparison of the entire operational life of the special-purpose vehicle.

In the literature [7], wear tests covering thousands of operating cycles were presented, which are extremely time-consuming and expensive. It was proposed to replace them with numerical methods. The presented method was tested and verified based on the results obtained from ball-on-disk sliding tests, carried out on different materials and under different conditions, in order to predict the wear profiles of the ball and the disk. In the literature [8,9], the authors presented interesting results concerning the abrasive wear of cutting tools of machining machines. The proposed methods were based on the use of the digital twin model. In the literature [10,11,12,13,14], on the other hand, the problems of abrasive wear of machine elements, which are caused by vibrations and several other adverse physical phenomena, were discussed. The author of the literature [15] presented the phenomenon of acoustic emission, which is defined as transient elastic stress waves spreading in the structure, generated due to the rapid release of energy due to internal changes in the structure or contact of asperities under load. The literature [16] describes the problem of using nanomaterials as elements protecting selected machine elements against abrasive wear. The literature [17,18,19,20] deals with abrasive wear of machine elements and the problems resulting from this phenomenon related to the increase in the temperature of surfaces exposed to friction. The literature [21,22] refers to models illustrating the mutual contact of two surfaces exposed to abrasion. The authors of the literature [23] described machine learning models about tribology. They also presented many interesting examples of the applications of these models. The literature [24] presented a comparative analysis of wear models to accurately predict the wear of selected machine parts and elements. In the literature [25,26], the authors presented a study of abrasive wear based on selected models. The studies were conducted on samples of high-grade structural steels.

The literature [1,2,27,28,29,30,31,32,33,34] deals with the application of mathematical models and methods in the theory of reliability about technical objects used in mechanical engineering. The aforementioned literature items were used by the author of this article to build and present the models illustrating the process of wear of selected elements of special vehicles.

### 2.1. Description of the Damage Process

The subject of the analysis in this article is the comparison of mathematical models illustrating the wear process of atypical elements, such as the following:

(a) The guides of the collecting and pressing plate in the plate-type garbage truck;
(b) The base of the pin fastening the eye of the container lifting cylinder of the loading device in a garbage truck with rear loading and plate compaction.

Two models were selected for this analysis:

(a) A model showing a damaged element, using the example of the guides of the collecting–pressing plate, where it is assumed that the guides in question are replaced with new ones.
(b) A model showing a damaged element, using the example of a base for securing the eye of a container lifting cylinder, where it is assumed that the element is being repaired, and the result of the repair is the complete restoration of the ability of the element to perform its functions.

In model (a), renewal is de facto replacement. There is no repair, and there is no need to consider whether the repair restored the ability of the element to work and to what extent. Each failure is equivalent to the need for a new element. After the element is replaced, the work continues as if from the beginning. If—after the element is damaged—we consider whether to repair the element or replace it with a new one, the time for replacing the element is usually short compared to the average time for repairing the element.

In the case of estimating the failure rate parameters (λ), statistical analysis was used to determine this value, including the maximum probability method. The justification for the selection of specific parameters is reflected in the calculations presented in the described example. λ—denotes the failure rate of the analyzed components.

In the analyzed example, the determination of the failure rate λ was based on an analysis of the company’s historical data regarding the vehicle class under study. This was due to the fact that the number of special vehicles tested, even in the case of a large company, does not constitute a statistically representative sample. Determining λ for special vehicles involved successively determining partial coefficients for a specific settlement period and then determining their arithmetic operating average. The assumed settlement period was the calendar year. The analyzed company is a municipal enterprise operating for over 30 years.

In model (b), we speak of repair as a consequence of the damage that occurred.

In reliability analyses, model (b1) is usually assumed, i.e., the complete restoration of the element’s ability to perform its functions. In the subject literature, it is often stated that after repair, the element is as good as new. The indicators and characteristics of the element’s reliability in model (a) and in model (b1) are the same, except that in model (a) we constantly need new elements, and in model (b1) there is a constant demand for repair. The replacement time (a) is synonymous with repair (b1), although the consequences of damage are different. It is worth realizing that if we are dealing with only one object, it cannot be repaired ad infinitum. At some point, the element or the entire object will have to be replaced [31].

Model (b2), i.e., partial restoration of the element’s ability to perform its functions, is very reluctantly considered in theory because it is usually a model that is complicated in nature. There are three reasons for this.

First, it is necessary to define the degree of restoration of this efficiency and to determine whether this restoration ability is constant or variable over time or with the number of repairs performed.

Let us further note that after the first repair, in the model under consideration, the element is not the same as it was at the beginning. The questions arise: How does the element change after the subsequent repairs? Are the changes characterized by any regularity? And if so, what kind? Obtaining answers to the questions posed is usually difficult. The point is to identify the regularities of a physical nature. If we try to write down these physical regularities in mathematical formulas, it will turn out that the notations will be very complicated; there will be many convolutions of functions and many conditional probabilities. This is the second reason.

The third reason is related to the possibilities of estimating these probabilistic characteristics. Their reliable estimation requires very rich statistical material and long and extensive studies. And this costs money. It is not uncommon for such studies to take so much time that the results would probably be useless. In such a long time, the technical object being tested would probably be modified, or the tested element [31].

Let us consider model (a)—the damaged element is replaced with a new one—and let us assume that the time of this renewal is negligible. This is the case, for example, in the studies of hydraulic motors used in special vehicles. The average operating time between adjacent failures of a hydraulic motor is quite long; there are several failures per year, on average. The average time to repair a hydraulic motor is about one hour.

**Figure 1:**
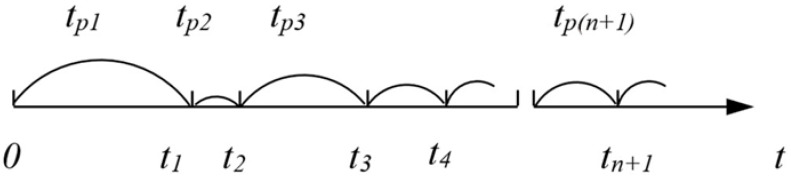
Graphic illustration of the damage process.

We can then treat the damage as an impulse, i.e., the replacement time is zero. The process of operating such an object from the reliability point of view is a stochastic impulse process, as shown in Figure 1.

To avoid overestimating the impact of varying operating conditions among garbage trucks, the authors considered garbage trucks from the same manufacturer, operated in the same city, and owned by the same company. According to the authors, collecting information on damage occurring in garbage trucks operating in similar terrain and climatic conditions significantly contributes to avoiding overestimating the impact of varying operating conditions among these vehicles. The only difference in this respect was the fractions of waste transported, which, due to their characteristics, had a greater or lesser impact on the occurrence of the described damage in vehicles such as municipal garbage trucks.

### 2.2. W.L. Smith’s Model of Renewal Theory

The described process is usually one of the first models considered in the theory of renewal. The foundations for this theory were laid by W. L. Smith [34], who published many works in this area in the fifties of the last century (1954–1959). Let us consider a set of homogeneous objects whose working times until failure are random variables with the same distribution F(t) for all objects. Let us assume that one of the objects started working and we will assume that this is the zero moment. The object worked for a certain time t_p1_ and at the moment t_1_ = t_p1_ (Figure 1) a failure occurred. The damaged object is immediately replaced by the next object that starts working. This object after working for the time t_p2_ failed at the moment t_2_ (Figure 1). Let us assume that the process of replacing damaged elements is continued.

Let us define a sequence of random variables:

(1) $$S_{n}=\sum_{i=1}^{n}t_{pi},\;n=1,2,\ldots ,$$

interpreted as moments of renewal of elements. The sequence $\left\{S_{n}\right\}$ is called the renewal stream. This stream is sometimes called the renewal process, but this term is not correct, because the time-axis representation of the sequence is not a stochastic process in the usual sense.

**Figure 2:**
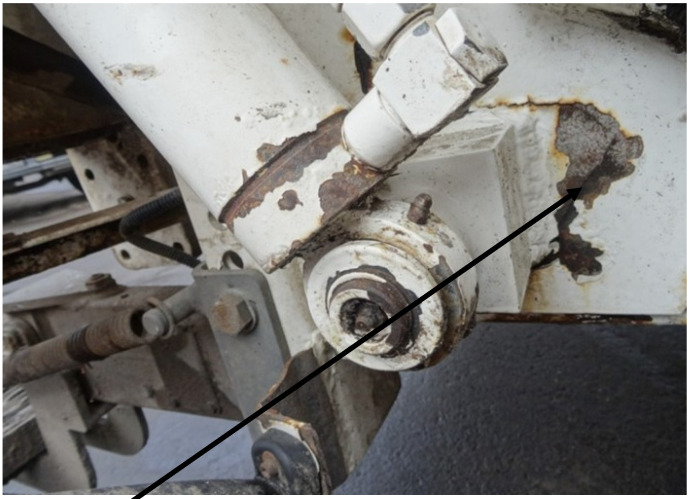
Base of the pin fastening the eye of the container lifting cylinder.

With reference to Figure 2, the calculations used to determine the presented curve used an approximation of experimental data, while its course was determined theoretically in order to better graphically present the described problem.

Let us denote by *N*(*t*) the number of failures in the time interval (0, t]. For a fixed t, the quantity *N*(*t*) is a random variable taking natural values. Let us define the probability distribution of this variable. Note that

(2) $$P\left\{N\left(t\right)\geq n\right\}=P\left\{S_{n}\right\}=P\left\{t_{p1}+t_{p2}+\ldots +t_{pn}<t\right\}=F_{n}\left(t\right)$$

where *F_n_*(*t*) is the distribution function of the random variable Sn. It is defined by the equation:

(3) $$F_{n}\left(t\right)=\int_{0}^{t}F_{n-1}\left(t-\tau \right)dF\left(\tau \right),\;F_{1}\left(t\right)=F\left(t\right),\;n=2,3,\ldots ,$$

Determining the function *F_n_*(*t*) is interesting from the operational point of view. An important issue is to obtain an answer to the question: What is the smallest necessary number of elements so that in the time interval [0, t] the renewal stream with probability (1 − α) does not have to end due to lack of spare elements? To obtain an answer to this question, it is necessary to find the smallest number n that satisfies the inequality:

(4) $$1-F_{n}\left(t\right)\geq 1-\alpha .$$

Finding the probability distribution of *F_n_*(*t*) for large n is generally troublesome. There are exceptions, however. For example, if the distribution of *F*(*t*) is exponential, then the distribution of *F_n_*(*t*) is the Erlang distribution of order n. Similarly, if the distribution of *F*(*t*) is the Erlang distribution of order r, then the distribution of *F_n_*(*t*) is the Erlang distribution of order nr. If the distribution of *F*(*t*) is a linear combination of Erlang distributions, then the Laplace–Stieltjes transform of the distribution of *F_n_*(*t*) is a rational function, and then the function *F_n_*(*t*) itself can be found by decomposing (here: dividing) the rational function into simple fractions.

#### 2.2.1. Example 1—Model Analysis Using the Example of the Base for Mounting the Eye of a Container Lifting Cylinder, Where It Is Assumed That This Element Is Being Repaired

**Figure 3:**
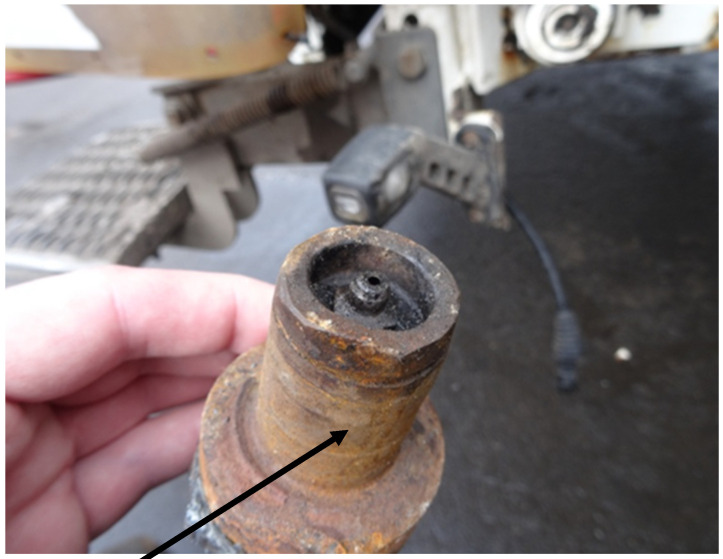
Pin connecting the hydraulic cylinder eye to the base.

The base of the pin fastening the lifting eye of the container cylinder of the loading device in a garbage truck with rear loading and plate compaction is an element that is exposed to tensile stresses during everyday operation. In this base, the forces from the weight of the container with the load, lifted to unload its contents into the garbage truck’s drop-in hopper, accumulate. This base is welded to the sheets of the outer casing of the garbage truck’s drop-in hopper; the base is shown in Figure 2, while the pin is shown in Figure 3.

The base shown in Figure 2 and the pin placed in it, shown in Figure 3, operate in very unfavorable conditions, because they are exposed to contamination generated around the loading area of the garbage truck, as well as to impacts from loading containers. The pin is equipped with a grease nipple to supply grease between the hydraulic cylinder eye and the external surface of the pin. Increased resistance to movement between the hydraulic cylinder eye and the pin also contributes to an increase in the value of forces acting on the pin base.

**Figure 4:**
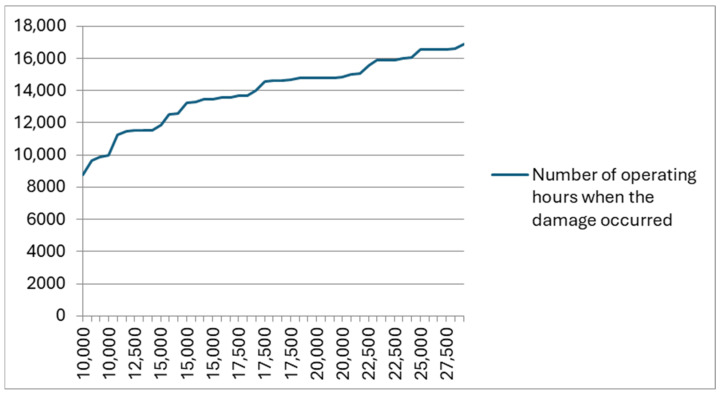
The course of damage to the base fastening the pin cooperating with the eye of the container lifting cylinder in a plate garbage truck, where the vertical axis shows the number of operating hours when the damage occurred, while the horizontal axis shows the total number of operating hours worked by the plate garbage trucks.

In the graph shown in Figure 4, the author presented an analysis of the damage course of the pin bases in 45 analyzed garbage trucks from the same manufacturer. These garbage trucks worked in similar conditions in different regions of Poland, collecting waste of a similar nature.

Based on the analysis presented in Figure 4, it results that the first damage in the form of torn-off bases fastening the pins occurs at the mileage of 9000 mth, after which the repair consists of replacing or strengthening the base. Depending on the thoroughness of the repair, the next damage to the base of the pin appears after a dozen or several dozen hours. It is worth noting that torn-off bases fastening the pins appeared in garbage trucks only a few years old, right after their warranty period.

**Figure 5:**
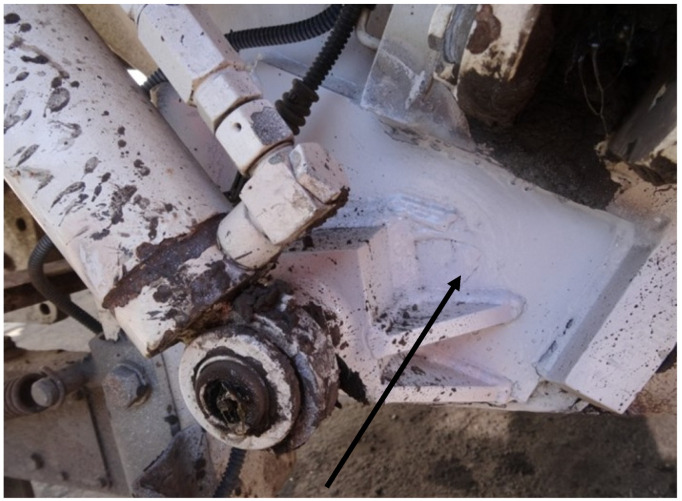
Reinforcement of the base for mounting the pin cooperating with the eye of the container lifting cylinder in a plate-type garbage truck.

In order to prevent the base of the pin securing the container lifting cylinder from being torn out, the author suggests reinforcing the socket of this base by welding a steel sheet around its base and additionally welding in side reinforcements, set at a 90° angle to the plane of the pin base plate. An example of the reinforcement described above is shown in Figure 5.

##### Mathematical Description of the Methodology for Solving the Presented Problem No. 1

Based on the analysis presented above, let us assume that the operating time of an element, which is the base fastening the pin cooperating with the eye of the container lifting cylinder in a plate garbage truck, has an exponential distribution with an average of α^−1^ = 350 h. Let us assume that we have seven such elements and we are interested in the probability of the event that these elements, when installed sequentially, will work for a total of at least 2600 h.

The damage moment of the seventh element S7 has an Erlang distribution of the seventh order with parameter α = 350^−1^. Considering formula (5), we calculate

(5) $$F(x)=1-\sum_{i=1}^{k-1}\frac{{\left(\lambda x\right)}^{i}}{i!}e^{-\lambda x},\;\;\;\;x>0,\;\lambda >0\;P\left(S_{7}\geq 2600\right)=1-F\left(2600\right)={\sum_{i=1}^{6}\frac{1}{i!}\left(\frac{2600}{350}\right)}^{i}\exp\left(-\frac{2600}{350}\right)=0,388.$$

**Figure 6:**
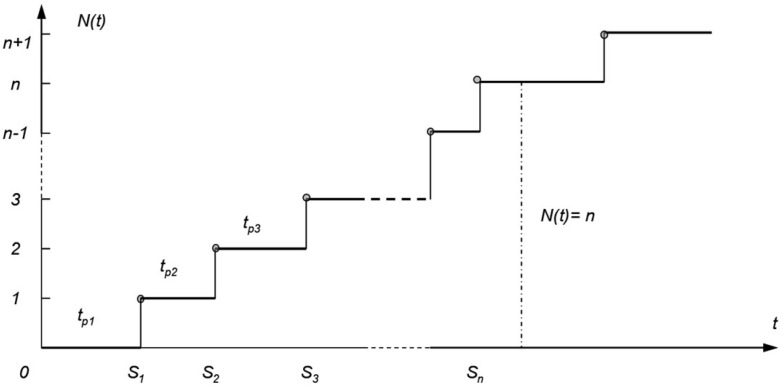
Implementation of the renewal process.

The number of failures in the renewal stream {Sn} in the interval (0, t) is called the renewal process and denoted by *N(t)*. This process for each t is a random variable taking non-negative integer values. It is assumed [1] that the realization of the renewal process is a left-continuous function, which is shown in Figure 6, where: F(x)—the distribution function of the random variable X, *P*—the probability of a random event.

To estimate the number of failures/renewals in other cases encountered in mechanical engineering, it is worth using the fact that the random variable *N*(*t*) has an asymptotically normal distribution with an expected value approximately equal to t/μ_1_ and a variance approximately equal to σ^2^t/μ_1_^3^, where μ_1_ is the expected value of the component operating time and σ^2^ is the variance of this random variable [32].

Hence, given the expected value estimates ${\bar{\mu }}_{1}$ and standard deviation $\hat{\sigma }$ we can estimate the interval random variable N(t) [29] so that the following equation is true:

(6) $$P\left\{\frac{t}{{\bar{\mu }}_{1}}-u_{\alpha /2}\frac{\hat{\sigma }\sqrt{t}}{{\bar{\mu }}_{1}^{3/2}}<N\left(t\right)<\frac{t}{{\bar{\mu }}_{1}}+u_{\alpha /2}\frac{\hat{\sigma }\sqrt{t}}{{\bar{\mu }}_{1}^{3/2}}\right\}=1-\alpha ,$$

where u_α/2_ is the order α/2 quantile of the standard normal distribution and α is the composite prior significance level.

Equation (6) is valid for large t.

If we are interested in the one-sided estimate from below, we are interested in the inequality:

(7) $$N\left(t\right)>\frac{t}{{\bar{\mu }}_{1}}-u_{1-\alpha }\frac{\hat{\sigma }\sqrt{t}}{{\bar{\mu }}_{1}^{3/2}}\;N\left(t\right)>\frac{2600}{350}-1645\frac{60\sqrt{2600}}{350^{3/2}}\approx 5.99$$

fulfilled with probability (1—α).

The above formulas are useful in operational practice, because we are usually interested in longer periods of operation of a technical object. We can therefore state that to ensure, with a probability of 0.95, continuous operation during 2600 h, the reinforcement of the base fastening the pin cooperating with the eye of the container lifting cylinder in a plate garbage truck should be repaired at least six times. Let us consider in more detail what the obtained result means. If we assume that the elements work 24 h a day, it means that when we speak of 2600 h, we are talking about over a year of operation of these elements. It follows that, on average, the base of the subject cylinder should be reinforced every 2 months. The result of the above calculations perfectly matches the operating data that the author obtained from workshops dealing with servicing this type of special vehicle. Because of the above, the model adopted above perfectly reflects the operational reality to which the analyzed technical object was subjected.

#### 2.2.2. Example 2—Analysis of the Model Using the Example of the Guides of the Collecting and Pressing Plate, Where It Was Assumed That the Guides in Question Were Replaced with New Ones

**Figure 7:**
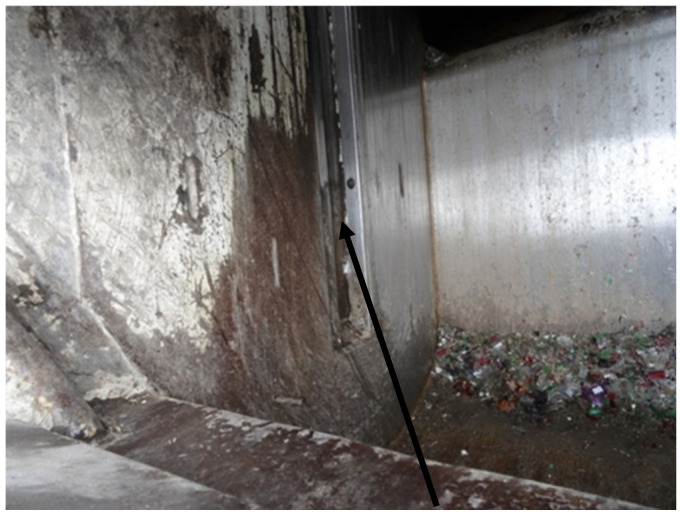
Guide of the collecting and pressing plate.

The guides of the collecting and pressing plate in a plate-type garbage truck are elements that serve to evenly guide the collecting and pressing plate both during the collection of waste from the garbage truck’s drop-in hopper to the main hopper and during the plate’s return to its lower position in the drop-in hopper. These guides are made in the internal parts of the rear housing of the garbage truck’s drop-in hopper; they are shown in Figure 7.

The guides of the collecting and pressing plate wear out naturally, as a result of friction and operation in very difficult and unfavorable conditions. Figure 7 shows one of the guides (marked with a red arrow) in a garbage truck designed to empty containers used to collect glass.

After loading a container with glass, cullet of various thickness is created from the glass in the lower container, which, when moving through the collecting and pressing plate, acts as an abrasive, wiping all cooperating surfaces, including the surfaces of the guides of the collecting and pressing plate.

**Figure 8:**
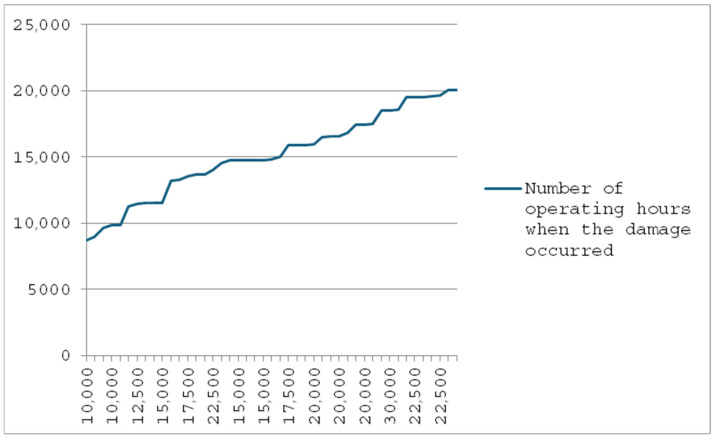
Damage (abrasion) to the guides of the collecting and pressing plates, where the vertical axis shows the number of operating hours when the damage occurred, while the horizontal axis shows the total number of operating hours worked by the plate garbage trucks.

Figure 8 shows a graph showing the results of tests on damage to the guides of the collecting and pressing plates, which were noted by the author from the repair and inspection books of 45 garbage trucks collecting waste of a similar nature.

Analyzing the graph from Figure 8, it can be seen that the first guide damages appear quite early, because already at the mileage of 8000–9000 mth, where the guides are regularly damaged. Only after their next replacement, i.e., up to a value of about 20,000 mth, does the graph have a linear course, while later, in the group of analyzed garbage trucks, successive damages start to appear again.

The problem of worn-out guides, based on the observations conducted by the author, results primarily from the material they are made of, which is a relatively low-quality plastic that is much more susceptible to abrasion than the guides used in older types of garbage trucks, where the guides were made of steel with a correspondingly lower hardness than the internal housing of the drop-in container. The older type of guides were much more durable and less prone to failure than those currently used, and to eliminate the problem of their wear, it would be necessary to return to the pattern from a dozen or so years ago, replacing the plastic guides with steel guides.

##### Mathematical Description of the Methodology for Solving the Presented Problem No. 2

Let us assume that the expected time of failure-free operation of an element, which is the guide of the collecting–compressing plate in a plate-type garbage truck, is μ_1_ = 230 h, and the standard deviation σ is 60 h. Let us estimate the necessary number of spare elements to ensure uninterrupted operation of the operated guides for the period t = 10,000 h.

Let us first assume the level of credibility. In most cases considered in mechanical engineering, the level (1 − α) = 0.95 is assumed, unless for some reason the considerations are special. For example, if the analysis is only superficial, the probability (1 − α) may be lower, in the order of 0.90. In safety considerations, on the other hand, the level of credibility is usually increased by assuming 0.99 or 0.995, or even 0.999.

From the tables of the standardized normal distribution, we read [30] (table U.II.2) u_0.95_ = 1645.

Using formula (7) we get

$$N\left(t\right)>\frac{10000}{230}-1645\frac{60\sqrt{10000}}{230^{3/2}}\approx 40.6.$$

We can therefore conclude that to ensure, with a probability of 0.95, continuous operation for 10,000 h, there should be a total of at least 41 elements (in this case, the mentioned guides).

Let us think a little more carefully about what the result means. If we assume that the elements work 24 h a day, this means that when we talk about 10,000 h, we are talking about more than a year of work of these elements. If the elements are expensive, then there is no point in buying 41 elements at once. Therefore, we need to solve the economic problem of how often to order the elements we are talking about here and in what quantity. This is beyond the scope of this article; however, having the patterns presented here, we can consider such a problem.

It is worth noting that in the literature [27] it has been shown that when

(a) elements have strictly increasing damage intensity,
(b) we are interested in time t less than the expected value of the element’s operating time, $t<\mu_{1}$.

Then, the following relationship is true:

(8) $$P\left(N\left(t\right)\geq r\right)\leq \sum_{i}\frac{{\left(t/\mu_{1}\right)}^{i}}{i!}\exp\left(-\frac{t}{\mu_{1}}\right).$$

It follows that the Poisson distribution gives an upper estimate of the probability of no fewer than r failures occurring in time [0, t] when the components have a strictly increasing risk function and when t is no greater than the expected operating time of a single component.

Let us recall that such a stochastic process is called a Poisson process with parameter λ$\left\{X_{t},t\geq 0\right\}$, which meets four conditions:

(a) $P\left(X_{0}=0\right)=1$;
(b) The process has independent increments, which means that for any 0≤ *t*_1_*< t*_2_ *< …< t_n_* random variables *X_t_*_1_, *X_t_*_2_ − *X_t_*_1_, *…*, *X_tn_* − *X_tn−_*_1_ they are independent;
(c) The process has uniform increments, i.e.,: X_t_ − X_s_ $\overset{d}{=}$ X_t−s_ (equality $\overset{d}{=}$ means equality of distributions);
(d) For any *t* > 0 the random variable X_t_ has a Poisson distribution with parameter λt, i.e.,:
  $$P\left(X_{t}=k\right)=\frac{{\left(\lambda t\right)}^{k}}{k!}\exp\left(-\lambda t\right),\;\;\;\;\;\;k=0,1,\ldots$$

It can be seen that the following equation is true:

(9) $$P\left(N\left(t\right)=r\right)=F_{r}\left(t\right)-F_{r+1}\left(t\right)\;\;r=0,1,2,\ldots$$

The fundamental role in the study of the renewal process is played by the function called the renewal function *H(t)*, which defines the expected number of failures up to time t. It is easy to see that

(10) $$H\left(t\right)=E\left[N\left(t\right)\right]=\sum_{n=1}^{\infty }nP\left[N\left(t\right)=n\right]=\sum_{n=1}^{\infty }n\left[F_{n}\left(t\right)-F_{n+1}\left(t\right)\right]=\sum_{n=1}^{\infty }F_{n}\left(t\right).$$

It can be proved [20] that this function satisfies the following integral equation:

(11) $$H\left(t\right)=F_{1}\left(t\right)+\int_{0}^{t}H\left(t-x\right)dF\left(x\right).$$

The important role of this function is explained by the fact that all the basic characteristics of the analyzed process are expressed with its help.

For example, the variance of the number of renewals is defined by the equation:

(12) $$\sigma^{2}\left[N\left(t\right)\right]=2\int_{0}^{t}H\left(t-x\right)dH\left(x\right)+H\left(t\right)-H^{2}\left(t\right).$$

Note that the average number of failures in time (*t*_1_, *t*_2_) is equal to *H*(*t*_2_) − *H*(*t*_1_).

Sometimes, instead of the renewal function *H*(*t*), the so-called renewal density function is defined by the equation:

(13) $$h\left(t\right)=H\prime \left(t\right).$$

It defines the average number of failures that will occur at moment t per unit of time, starting from moment t, provided that this moment is sufficiently small.

Considering formula (11) and formula (13), it can be seen that the following relation is true:

(14) $$h\left(t\right)=f_{1}\left(t\right)+\int_{0}^{t}h\left(t-x\right)f\left(x\right)dx.$$

Let’s move on to the Laplace–Stieltjes transforms. Then—taking into account the above relationship—we have the following equality:

$$h^{*}\left(s\right)={f_{1}}^{*}\left(s\right)+h^{*}\left(s\right)f^{*}\left(s\right)$$

which gives:

(15) $$h^{*}\left(s\right)=\frac{f_{1}^{*}\left(s\right)}{1-f^{*}\left(s\right)}.$$

Let us now consider the basic reliability characteristics in cases where the distribution of operating times between adjacent faults is exponential, Gaussian, and described by a gamma distribution.

If the distribution of component operating times is exponential, then

(16) $$P\left(N\left(t\right)=r\right)=\frac{{\left(\lambda t\right)}^{r}}{r!}\exp\left(-\lambda t\right).$$

So, it is a Poisson process. Next:

(17) $$H\left(t\right)=\lambda t\;\;h\left(t\right)=\lambda .$$

It is the only stationary and consequence-free process (a process that is characterized by the absence of memory). The absence of memory for a process means that the probability of a certain number of failures occurring in a given time interval does not depend on how many failures have occurred up to that time and how. In particular, the absence of memory means the mutual independence of the occurrence of a certain number of failures in non-overlapping time intervals.

If the distribution of the working times of the elements is approximated by the normal distribution and furthermore σ << μ_1_, then

(18) $$F_{r}\left(t\right)=\Phi \left(\frac{t-r\mu_{1}}{\sigma \sqrt{r}}\right),\;\Phi \left(x\right)=\frac{1}{\sqrt{2\pi }}\int_{-\infty }^{x}\exp\left(-\frac{x^{2}}{2}\right)dx.$$

The renewal function in this case is defined by the formula:

(19) $$H\left(t\right)=\sum_{r=1}^{\infty }\Phi \left(\frac{t-r\mu_{1}}{\sigma \sqrt{r}}\right)$$

while the renewal density is determined by the relationship:

(20) $$h\left(t\right)=\sum_{r=1}^{\infty }\frac{1}{\sigma \sqrt{2\pi r}}\exp\left(-\frac{{\left(t-r\mu_{1}\right)}^{2}}{2r\sigma^{2}}\right).$$

**Figure 9:**
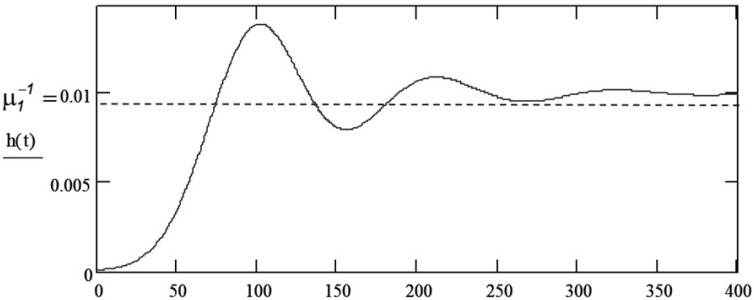
Example of the renewal density function h(t).

An example of this function is shown in Figure 9. This is a characteristic form of damped oscillation.

If the distribution of element operating times is a gamma distribution, i.e.,:

$$f\left(t,\alpha \right)=\frac{\lambda^{\alpha }}{\Gamma \left(\alpha \right)}t^{\alpha -1}\exp\left(-\lambda t\right)$$

then

$$f\left(t,\alpha n\right)=\frac{\lambda^{n\alpha }}{\Gamma \left(n\alpha \right)}t^{n\alpha -1}\exp\left(-\lambda t\right)$$

and then the renewal density function:

(21) $$h\left(t\right)=\sum_{n=1}^{\infty }\frac{\lambda^{n\alpha }}{\Gamma \left(n\alpha \right)}t^{n\alpha -1}\exp\left(-\lambda t\right).$$

If the shape parameter is a natural number m, which means that the gamma distribution becomes the Erlang distribution, then the series in formula (19) sum up and the renewal function is given by formula [32]:

(22) $$H\left(t\right)=\frac{1}{m}\sum_{j=1}^{m-1}\left(\lambda t+\frac{\varepsilon^{j}}{\varepsilon^{ji}-1}\exp\left(\lambda t\left(\varepsilon^{j}-1\right)\right)\right),\;m\in N$$

where N is the set of natural numbers, and

(23) $$\varepsilon =\cos\frac{2\pi }{m}+i\sin\frac{2\pi }{m}\;\varepsilon =1074\approx 1$$

For other distributions used in reliability theory, the function *F_n_*(*t*) is not expressed in finite form and then the calculations are much more difficult.

The model used in example 2 provides the maintenance services (service) with an answer as to how many guides, on average, will be damaged during the year. Thanks to this information, it is worth taking care of the delivery of the necessary spare parts to the warehouse used by service technicians in a given company operating special vehicles in advance. As mentioned earlier, there is no point in buying 41 elements at once because, firstly, it would be too much of a financial burden for the company and, secondly, there would be a problem with storing these parts. Therefore, having a ready calculation algorithm in the presented mathematical model, the delivery of damaged elements should be carried out successively, depending on the needs and the number of special vehicles operated in a given time.

## 3. Discussion

The mathematical models presented in this article are a very useful tool for maintenance services, which provides an answer in the form of estimated damage occurrence in selected elements of special vehicles with a fairly high probability. Thanks to the adopted models and following the examples where these models were applied and about specific numerical data that were taken from operational practice, the following answers were obtained.

(a) How long should the repair of an element be carried out, the properties of which return to their nominal values after the repair?
(b) How many pieces of consumables should be replaced with new ones within a given time? In this case, it is also worth considering the successive delivery of these items, knowing how many of these items will be damaged, on average, within a given time.
  The author compared the following models, assuming that
  (a) the elements work until the first failure,
  (b) the elements are repairable, and after the repair they regain their properties and nominal technical parameters.

After selecting the models, it is worth answering the following questions:

(a) Is the wear of selected components of special vehicles based on the analysis of the adopted mathematical models consistent with operational practice?
(b) Which model is more appropriate for the nature of the wear of a given element, and in what context?

The answer to the above questions results primarily from the specifics of the work of special vehicles, and thus from the working conditions to which the elements analyzed in them are subjected, which are, the guides of the collecting–pressing plate and the base fastening the eye of the container lifting cylinder. It is worth mentioning here that the author classified the aforementioned guide as an element that is not repairable and after damage it should be replaced with a new one. This results primarily from the specifics of the wear process, which ultimately leads to a reduction, as a result of abrasion, of the geometric dimensions of this guide, and thus prevents linear guidance of the collecting–pressing plate in the loading device of the plate garbage truck. Therefore, the model providing the answer to how many guides will be used up on average in a year and which ones should be purchased later is important information for maintenance service employees, in order to replenish the warehouse stocks of these elements. Of course, as already mentioned, there is no point in buying the full quantity of guides at once, because storing them would be problematic, but it is worth applying the principle of sustainable spare parts management and having guides in stock in sufficient quantity for 2 or 3 months and then making further purchases.

In the case of problems resulting from the detachment of the bases securing the container lifting mechanism cylinders in a plate garbage truck, the model used in this article provides very important information on how often, on average, the base of the cylinder will be detached. In this case, an annual period of operation of a given special vehicle was also assumed. The model used about the analyzed bases of the cylinder mounting is a very important indicator for maintenance services, especially assuming that a given waste disposal company has several garbage trucks with plate compaction, and their work is identical. The results obtained from this model will allow the organization of the work of maintenance services to work more rhythmically, and therefore more effectively. In addition, the tool in the form of the aforementioned model will allow for reducing the queue of damaged garbage trucks waiting for repair.

In this article, the authors have avoided presenting a further analysis of the sensitivity of the models to changes in the parameters used in the described examples. This decision was made due to the limited space available in this article; however, the authors are planning a subsequent publication on this topic. Regarding the issue of restoring the functional characteristics of a technical object, such as a special-purpose vehicle, i.e., replacement versus regeneration, the primary goal is to achieve full technical efficiency of the technical object.

Companies specializing in urban cleanup rely on this premise. Rationale: In times of shortage of spare parts, including materials such as sheet metal or welding electrodes, it is faster and more cost-effective to replace a given component with a new one. However, if the component is not commercially available, then regeneration is the only option, i.e., the full or partial restoration of the damaged component’s nominal parameters and functional characteristics.

The main obstacle to developing a discussion aimed at a detailed comparison of the obtained results with existing and available literature is the lack of studies in this area. The literature analyzed by the authors of this article clearly indicates a research gap in the field of special vehicle operation. It is worth mentioning that the research described in the examples in this article used actual operational data to ensure that the obtained results reflected the actual problems arising from the operation of a fleet of special vehicles. The authors consulted the obtained results with maintenance services, as well as with the manufacturers and service technicians of special vehicles—garbage trucks, whose selected components subject to damage were analyzed in the described examples. The results of these consultations indicate the accuracy of the results obtained by the authors and are presented in both examples.

## 4. Conclusions

In summary, the author would like to point out that all data analyzed based on the cited models were operational data, having coverage in the reality in which technical objects in the form of special vehicles operate. The results obtained from the analyzed models match very well the operational practice that was analyzed to build this article.

The models and their corresponding dependencies presented in this article are used, among others, in the description of the reliability of many mechanical elements, as well as hydraulic components, including hydraulic motors. They are also often used in the modeling and analysis of the reliability of mechanical objects in cases where their reliability is very high in the sense that the operating time of the objects is very long while the repair time is relatively short. Unfortunately, there are not many such mechanical objects in mechanical engineering.

Hydraulic motors operating in the analyzed technical objects, such as special vehicles, are honorable exceptions in this respect. Their operating process from the reliability point of view is sometimes described both by process models with negligibly small renewal, as well as by renewal process models with finite renewal time.

The results of the tests conducted clearly indicate the correct selection of the elements constituting the described hydraulic transmission, which replaced the drive shaft with Cardan joints. The characteristics of the selected hydraulic elements, including the hydraulic motor and the main multi-section hydraulic pump, ideally matched the characteristics of the work of the WUKO ZM-260 mechanical sweeper and its modified model ZM-260V. In the future, the authors intend to conduct further research aimed at modernizing the drive systems in special vehicles such as mechanical sweepers and others. The aim of this research is to reduce the amount of energy consumed by the sweeper, including fuel, reduce noise, and increase durability and reliability during operation.

The practical implications for maintenance services, after analyzing the text of this article, should be indications of the time when a given component fails, as well as the number of failures within a given time period. With this data, and with full documentation of overhauls and repairs, maintenance services can estimate when a given component will wear out and require replacement or reconditioning. Furthermore, with information on the operating time of a given component, maintenance services can secure the appropriate number of necessary components to avoid taking a damaged vehicle out of service.

The inclusion of a dedicated research section devoted to study limitations (including data limitations and model assumptions) was partially omitted by the authors due to space constraints in this article. However, the authors will certainly publish their findings in this area as well.

Finally, it is worth noting that the authors did not actually compare the results obtained in the field of repair and renovation of special vehicles with other studies. The rationale: there is a lack of relevant articles and studies relating to the maintenance of fleets of special vehicles, such as urban cleaning vehicles, including waste collection. The scarcity of such studies constitutes an obstacle to comparing the results obtained in the field of repair and renovation of special vehicles.

## Data Availability

The original contributions presented in this study are included in the article. Further inquiries can be directed to the corresponding authors.
